# Public attitudes toward the use of technology to create new types of animals and animal products

**DOI:** 10.1017/awf.2023.38

**Published:** 2023-06-06

**Authors:** Erin B Ryan, Daniel M Weary

**Affiliations:** Animal Welfare Program, Faculty of Land and Food Systems, University of British Columbia, Vancouver, BC, Canada

**Keywords:** Animal welfare, bioethics, environmental ethics, gene editing, genetic modification, farming systems

## Abstract

Philosophers have used thought experiments to examine contentious examples of genetic modification. We hypothesised that these examples would prove useful in provoking responses from lay participants concerning technological interventions used to address welfare concerns. We asked 747 US and Canadian citizens to respond to two scenarios based on these thought experiments: genetically modifying chickens to produce blind progeny that are less likely to engage in feather-pecking (BC); and genetically modifying animals to create progeny that do not experience any subjective state (i.e. incapable of experiencing pain or fear; IA). For contrast, we assessed a third scenario that also resulted in the production of animal protein with no risk of suffering but did not involve genetically modifying animals: the development of cultured meat (CM). Participants indicated on a seven-point scale how acceptable they considered the technology (1 = very wrong to do; 7 = very right to do), and provided a text-based, open-ended explanation of their response. The creation of cultured meat was judged more acceptable than the creation of blind chickens and insentient animals. Qualitative responses indicated that some participants accepted the constraints imposed by the thought experiment, for example, by accepting perceived harms of the technology to achieve perceived benefits in reducing animal suffering. Others expressed discomfort with such trade-offs, advocating for other approaches to reducing harm. We conclude that people vary in their acceptance of interventions within existing systems, with some calling for transformational change.

## Introduction

Animal agriculture faces moral challenges, including concerns about animal welfare. In response, policy-makers and entrepreneurs sometimes suggest technological solutions that can themselves pose ethical concerns (Murphy & Kabasenche 2018; Dryzek *et al.* 2020). Technologies such as CRISPR (clustered, regularly interspaced, short palindromic repeats), that allow changes to the genomes of organisms used in agriculture, are seen as a ‘game-changer’ by proponents (Schultz-Bergin 2018; p 219), but public attitudes to such technologies can be negative depending upon the context. For example, a study in the United Kingdom found that the majority of people were positive about the use of “genome editing to produce more nutritious crops”, but there was “much less support for the genetic modification of animals for food” (van Mil *et al.* 2017). This conclusion is consistent with previous studies in North America (Hoban 1998; Cuite 2005) that found that genetic engineering of animals is viewed as the most negative of various food technologies, even when compared to the use of pesticides and hormones (Henson *et al.* 2008). Public attitudes also vary by case; recent research on North American and European participants found that gene editing farm animals to increase muscle mass and productivity was viewed as less acceptable than applications to increase disease resistance (Busch *et al.* 2022).

The perceived motivation in developing the technology also affects public views. For example, when genomic technology was described as reducing harm to animals or the environment it was viewed as more positive than when described as improving profitability (Ritter *et al.* 2019), suggesting that people are more receptive of technologies perceived to provide more than just economic benefits. A study on the acceptability of genetic technologies applied to agricultural animals found support for applications that enhanced animal welfare (e.g. via increased disease resistance), but not for applications that simply increased food production (Naab *et al.* 2021). In addition, people appear to be more accepting when they believe that the suggested intervention is a viable solution and that alternatives are not as likely to succeed in achieving the desired end (Schultz-Bergin 2014).

The examples considered in recent studies of public attitudes have focused on genetic applications such as creating hornless cows, disease resistance, and increasing muscle mass; these examples are relatively innocuous compared to examples discussed in the philosophical literature. The latter has focused on examples of disenhacement that remove biological capacities to allow animals to better cope with aversive environments (Sandøe *et al.* 1999; Thompson 2008; Palmer 2011; Murphy & Kabasenche 2018).

These more contentious examples have been explored using ‘thought experiments’ that involve a careful examination of the ethical consequences of constrained scenarios (Brun 2018). Briefly, a thought experimenter considers a scenario related to the moral permissibility of an action. By altering and excluding certain factors in test cases, thinkers are able to probe intuitions and test arguments in order to carefully consider individual factors that matter. After reasoning about the relevant details, the experimenter then puts forth a moral argument. Using this approach, Thompson outlined arguments in support of technology to dis-enhance animals, so as to prevent suffering (Thompson 2008), based upon the premise that many animals would otherwise suffer as a result of living in systems that they are poorly suited to. However, as Thompson (2008) noted, intuitions persist that this approach to dealing with animal suffering is wrong. Some philosophers have argued that a focus on preventing animal suffering is too narrow, as it fails to consider other issues that hold moral weight, such as animal dignity, integrity, positive welfare, instrumentalisation and exploitation, and how animals are represented in agriculture (Bovenkerk 2020). There is a rich body of philosophical literature that explores these other issues (Sandøe *et al.* 1999; Thompson 2008; Palmer 2011; Harfeld *et al.* 2016; de Graeff *et al.* 2019; Bovenkerk 2020).

The aim of this paper was to use the contentious examples considered in earlier thought experiments to provoke responses from lay participants, as a way of understanding their willingness to accept technological interventions to address welfare concerns. As Schultz-Bergin (2018) points out, the speed at which genetic technologies are being developed may outpace our ability to ethically reflect on the ways they might change our relationships to the animals we raise for food, so efforts to engage with the public and to empirically ground their input on a broad range of technologies used to transform animals is warranted. We used an online survey to ask public participants to respond to one of three scenarios that involved the use of technologies attempting to generate animal products while causing less suffering than existing methods of production. Two contentious examples had been discussed in philosophical literature. The first was creating a strain of blind chickens to reduce problems of feather-pecking and cannibalism in commercial flocks (Thompson 2008), a transformation possible through both traditional breeding and genetic modification; Thompson suggested this as an example, “emblematic of proposals put forth to reduce or eliminate suffering in production environments” (Thompson 2008; p 306). The second was the creation of insentient farm animals, suggested as another way to eliminate suffering in farm animals (Gifford 2002); the feasibility of this transformation is less clear, but it provides an interesting case to explore the limits of what genetic modifications to farm animals people may consider acceptable. Thompson encourages casting a wide net “to discuss possible scenarios well in advance of our ability to identify specific technological applications… that might lead to their realisation” (Thompson 2008; p 307). For comparison, we also included a third scenario: creating cultured meat (a technology under development with products already approved for sale in Singapore; Ketelings *et al.* 2021). This last scenario was included as a technological attempt to create an animal product without causing animal suffering, but also without creating a new dis-enhanced strain of animals. Thus, all three scenarios involve technology intended to reduce animal suffering, compared to other (i.e. unchanged) farmed animals, with the blind and insentient modifications changing the nature of this strain of animals to avoid harm. We expected participant support for animal agriculture, whether they believed the product was available on the market, and whether they believed that the technology respected the dignity of life would affect responses, so these variables were also included in our quantitative model of acceptance.

Our scenarios were structured to help us determine if lay participants would be willing to accept the constraints of the thought experiments and if the options offered were consistent with their values. Much of the research to date seeking public input on animal welfare issues uses framing that specifically invokes trade-off thinking within a constrained scenario (e.g. choices offered to participants exist within the *status quo* of industrial agriculture). This too was how our scenarios were framed, so we expected that some respondents would indeed engage in trade-off thinking. However, the contentious nature of these scenarios was expected to create unease on the part of some participants, increasing the chance that some would reject the choices offered. To understand the rationale of our participants, we asked them to report on the reasons behind their choices.

## Materials and methods

We studied US and Canadian participants over the age of 18 years. Participants were informed that data collected would not personally identify them, that they were free to leave the survey at any point and were provided information about storage and privacy of the survey data. Participants provided their consent to participate before beginning the survey. This study was approved by the University of British Columbia’s Behavioural Ethics Review Board (ID #H19-02069).

### Survey design

The full text of the survey along with SAS and NVivo files are available at https://doi.org/10.5683/SP3/J6JG9D. All participants began by answering a series of demographic questions (age, gender identity, income, whether they had children, highest level of education); these responses were collected to better describe our survey sample, followed by three questions aimed at understanding participant support for agriculture, including whether they felt that animal agriculture harmed animals or the environment (all measured using a five-point scale; 1 = definitely yes; 5 = definitely not).

Participants were then randomly assigned to one of the three scenarios. Each scenario involved a technological application that attempted to generate animal products while causing less suffering than existing methods of production: (1) the use of genetic modification to blind chickens to reduce injuries and death associated with feather-pecking; (2) the use of genetic modification to eliminate animals’ ability to experience emotions (i.e. creating insentient animals), thus eliminating suffering associated with transport, handling, etc; and (3) the creation of cultured meat to produce animal products without raising sentient animals (i.e. using genetic material from an animal to create an edible animal product). Acceptability of each scenario was rated using a seven-point scale (1 = very wrong to do; 7 = very right to do). Results throughout the paper referencing responses to this question refer to participant ‘support’ for each scenario. For the first two scenarios, we indicated that new strains would be ‘created’ through genetic means to address animal welfare issues, with the intention to convey that existing individual animals would not be changed but rather that new modified animals would be born. To better understand participant responses, this Likert-type response was followed by an open-ended question that asked participants to explain their answer in a sentence or two. Table 1 shows the wording for each scenario.

**Table 1.** Wording of scenarios. Each participant was randomly assigned to one of the three scenarios. Each scenario proposes a technology intended to create new types of farm animals or meat products

Scenario	Wording
Blind Chickens (BC)	Chickens housed in groups can peck one another, sometimes resulting in injury or even death. To deal with this problem, farmers generally remove part of the beak of young chicks without the use of anesthetic (i.e. pain-control)
	Alternatively, it is possible to use genetic modification to create chickens that are born blind. Blind chickens live similarly to sighted chickens but are less likely to engage in harmful pecking, eliminating the need to partially remove their beaks
	In your opinion, creating blind chickens would be (7-point scale)
	In a sentence or two, please explain your answer (Text box)
Insentient Animals (IA)	Farm animals can experience a number of unpleasant emotions, such as pain from castration, discomfort associated with restrictive housing, and fear associated with transport and handling
	To deal with these problems, some have suggested genetically modifying farm animals to create ’insentient’ strains that are born without the capacity to feel pain, fear, or pleasure
	In your opinion, creating insentient animals would be (7-point scale)
	In a sentence or two, please explain your answer (Text box)
Cultured Meat (CM)	Farm animals can experience a number of unpleasant emotions, such as pain from castration, discomfort associated with restrictive housing, and fear associated with transport and handling
	To deal with these problems, it is possible to use genetic engineering to create cultured (lab-grown) meat that is the same as that from animals raised for food but without concern for animal suffering
	In your opinion, creating cultured meat would be (7-point scale)
	In a sentence or two, please explain your answer (Text box)

A question to check participant attention followed, adapted from Oppenheimer *et al.* (2009) to fit the context of our survey. Participants were also asked to indicate the perceived beneficiary of the scenario (farmers, consumers, the animals, the agricultural industry, no one, or other). Two questions then used a five-point scale (1 = definitely yes; 5 = definitely not) to assess whether participants: (1) considered that the proposed solution respected the dignity of life; and (2) thought the proposed solution was currently available on the market. Participants then answered Haidt and Graham’s Moral Foundation questionnaire (2007) and responses were used to create three psychological constructs known to influence attitudes: Fairness, Care, and Sanctity (not analysed but available in the full text of survey and data analysis files). The survey closed with an additional demographic question on religious affiliation (not analysed) and diet (i.e. whether participants were omnivores, vegans, or vegetarians).

### Data collection

A demographically matched sample of participants was recruited through Dynata (www.dynata.com) from December 10, 2019, to January 7, 2020; sampling was based on US (2018) and Canadian (2016) census data for age, gender, and income. For a completed survey, participants were paid $US1.50. The survey was built and hosted on Qualtrics (www.qualtrics.com).

A total of 1,069 participants completed the survey. Of these, 886 passed the attention check. Median time to completion of the survey was 418 s. The 87 participants who completed the survey in less than half the median time (i.e. < 214 s) were excluded from the analysis, as were 52 participants who provided just one-word responses to the open-ended question, resulting in a final sample of 747 responses.

### Analysis

All participant data are available online as part of the full data set (https://doi.org/10.5683/SP3/J6JG9D).

### Quantitative

We began our analysis by assessing univariable associations (using Proc GLM in SAS Studio) between the participants’ attitude to the proposed technology (i.e. their response to the question “In your opinion, do you think it’s right or wrong to [use this technology] to deal with these problems?”) (coded as 1 = very wrong and 7 = very right) and each of the covariates: country of residence (Canada vs. US), gender (dichotomised as female vs. any other gender), age (treated as continuous with four levels), education (treated as continuous with eight levels), income (treated as continuous with 12 levels), if they had children (yes or no), if they considered animal agriculture to harm animals, or to harm the environment, and whether they were supportive of animal agriculture (all three coded as 1 = ‘definitely yes’ through to 5 = ‘definitely not’), and if they consumed meat (yes or no). We followed by assessing if any covariates with significant (*P* < 0.05) univariable associations were correlated; no correlation exceeded *r* = 0.5.

We then ran a full model testing the effect of scenario and including all covariates with significant univariable associations, and first-order interactions between scenario and the covariates (to determine if support for scenario varied in relation to the covariate). Significant interactions were then explored by repeating the analysis separately by scenario.

Participants also responded to a series of questions designed to explore the reasons for their overall attitude to the scenarios: the perceived beneficiary of the scenario (dichotomised as including ‘animals’ or not), if the scenario respected dignity of life, and if the scenario was currently available on the market (both varying from 1 = ‘definitely yes’ to 5 = ‘definitely not’). Given that these questions were all asked relative to the scenario, these effects were also analysed separately by scenario following the same procedure used to investigate the significant interactions.

### Qualitative

Participants were asked an open-ended question (i.e. “In a sentence or two, please explain your answer”) following the question regarding the acceptability of each scenario. We were specifically interested in how the method of presenting choices to participants (i.e. not presenting them with alternatives outside of the scenario) influenced participant choices; namely, if they engaged in the trade-offs inherent to the scenario, or if they rejected both options offered. We thus coded responses using two *a priori* codes: ‘Engaged in trade-off’ and ‘Rejecting choices’ (i.e. rejecting trade-offs offered in the presentation of scenarios). We also used five other themes (Naturalness, Consumption (of foods altered by technologies), Concerns related to technology, Opposition to technology, and Moral concerns) identified in previous research on attitudes to genetic modification of farm animals (Ritter *et al.* 2019); these themes are well described in the literature so results are not presented below, but coding can be seen in data (https://doi.org/10.5683/SP3/J6JG9D).

From our data set of 747 responses, we selected every 15th response (a total of 50 responses) to initially read through. Two coders (EB Ryan and C Cordoso) independently read the selected responses and met to compare and discuss how comments were coded and to agree upon final codes for the dataset. The entire dataset was then read by the same two individuals again and independently coded using the agreed upon codes. These individuals then discussed findings of the complete dataset and resolved any discrepancies in coding. Responses could contain multiple themes, and generic qualitative methods employing theoretical analysis was used to approach and understand the data (Percy *et al.* 2015). Quotes presented in the results were selected as best representing the data.

## Results and Discussion

### Sample description

Of the 747 participants in our final data set, 410 were Canadian, 381 were female, 441 had children, 416 had completed less than four years of post-high school education, 492 were at least 45 years old, and 675 were meat-eaters. Approximately equal numbers of participants were allocated to each of the three scenarios (Table 2).

**Table 2.** The number of participants in relation to key demographic categories: country of residence, participant age, gender, education, and dietary preference. Numbers are shown separately for participants allocated to each of the three scenarios (blind chickens, insentient animals, and laboratory meat)

		Scenario	
	Blind (n = 257)	Insentient (n = 248)	Lab meat (n = 242)
Country of residence			
US	112	112	113
Canada	145	136	129
Age			
18-29	29	29	20
30-44	71	47	59
45-59	78	74	73
Over 60	79	98	90
Gender			
Female	134	120	127
Other^1^	123	128	115
Education			
Completed 4-year degree or higher	116	105	110
Completed 2-year degree or less	141	143	132
Diet			
‘I eat meat’	232	230	213
Other^2^	25	18	29

1 361 of these participants identified as male, one identified as gender variant/non-conforming, one as transgender, and one as ‘not listed’

2 This category includes all responses other than ‘I eat meat’

### Quantitative results

Participants expressed negative attitudes toward the blind chicken (mean [± SEM] acceptability of 2.5 [± 0.1]; where 1 = very wrong to do, and 7 = very right to do) and insentient animal scenarios (2.6 [± 0.1]), and ambivalence towards the laboratory meat (3.7 [± 0.1]). To some extent, the insentient animal and cultured meat scenarios can be considered similar, as they both result in the production of animal protein without risk of animal suffering. From this perspective, it seems counter-intuitive that the respondents in this study would perceive the insentient animal scenario more negatively. Further work is required to understand this result, but it is possible that respondents were perceiving morally relevant distinctions between these cases. For example, participants may have been concerned that the insentient animals were also unable to experience pleasure or other positive emotions, and this inability to experience positive states may be eliciting moral concern. Research on robot ethics suggests that physicality is also important. For example, Darling (2016) explored attachments to virtual life forms with robots that can be physically engaged with, concluding that people are more likely to relate to, and form emotional attachments with, those who inhabit physical space and have characteristics we recognise as belonging to a sentient life form. Thus, physicality can affect our assessment of whom we owe moral consideration (Darling 2016; Heller 2016), and in the current study the physical nature of the insentient animals may have provoked greater moral concern than the cell culture.

**Figure 1.**
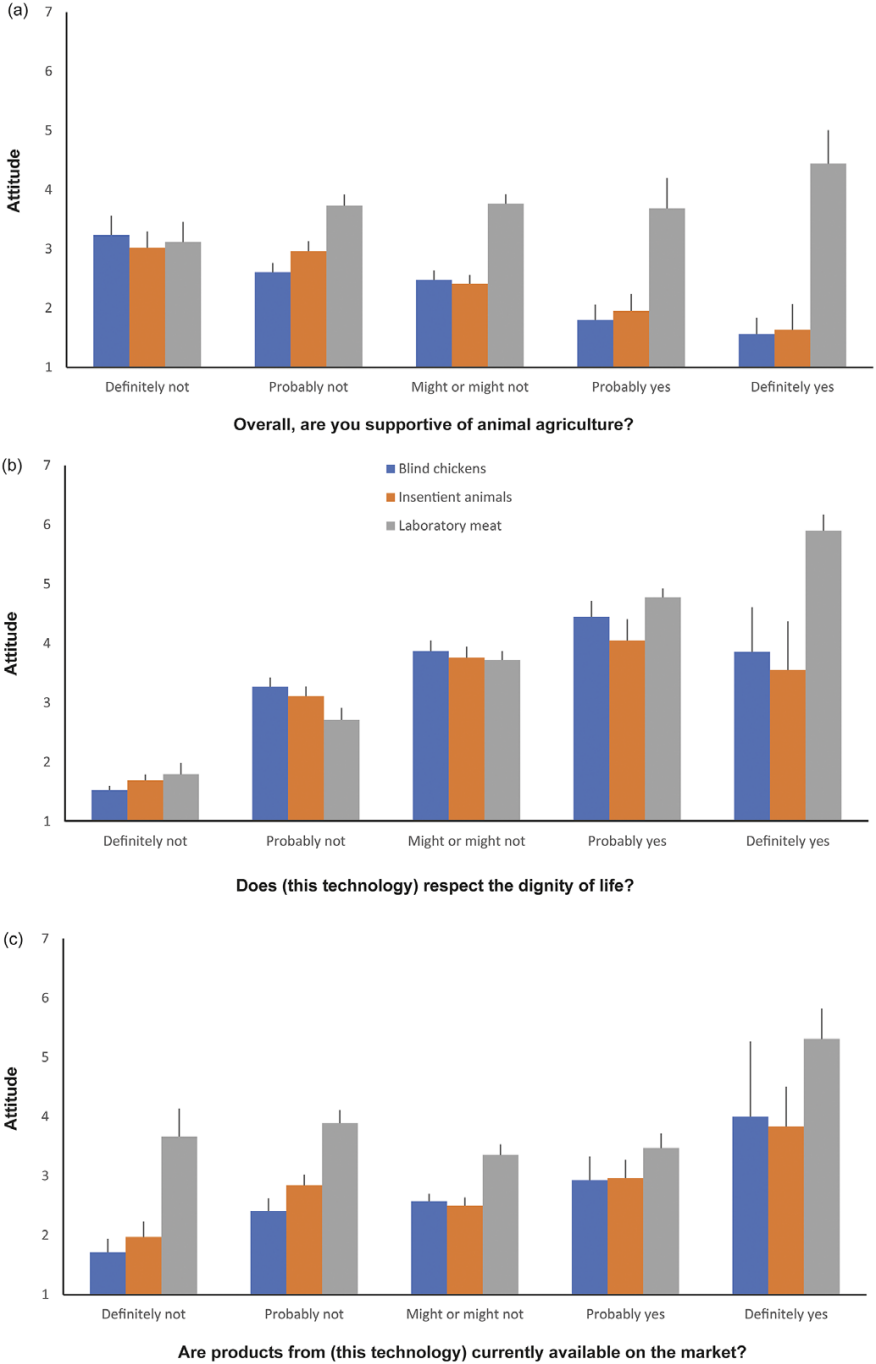
Mean (± SEM) participant responses to the question, “In your opinion, do you think it’s right or wrong to [use this technology] to deal with these problems?”, varying from 1 = ’Very wrong’ to 7 = ’Very right.’ Responses are shown separately by scenario (i.e. each of three proposed technologies: blind chickens, insentient animals and creating laboratory meat), and in relation to whether participants (a) expressed support for animal agriculture, (b) felt that the technology respected the dignity of life, and (c) believed that the technology was currently available on the market.

We found univariable associations between participant support for scenario and gender, if they had children, support for animal agriculture, and if they ate meat, so our final model included all four covariates. However, in the full model, the effects of gender and children (and interactions between these variables and scenario) were not significant, so these are not reported further. We did find an interaction between scenario and support for animal agriculture (*F*_2,732_ = 9.66; *P* < 0.0001), and some evidence of an interaction between scenario and whether participants ate meat (*F*_2,732_ = 2.84; *P* = 0.0591), so these covariates were explored separately by scenario. For participants assigned to the blind chicken and insentient animal scenarios, those more supportive of animal agriculture, were less supportive of the technology (slope = –0.43, SE = 0.09, t_254_ = –4.58; *P* < 0.0001; and slope = –0.38, SE = 0.09, t_245_ = –4.03; *P* < 0.0001, respectively). For participants assigned to the laboratory meat scenario, we found no evidence of an association between support for animal agriculture and support for the technology (slope = 0.15, SE = 0.11, t_236_ = 1.40; *P* = 0.1641) (Figure 1[a]). We suggest that participants most supportive of animal agriculture were more engaged with the questions this study raised, and thus more likely to express a firm opinion, than those less supportive of agriculture or those generally ambivalent toward agricultural practices. These results also suggest that people most supportive of animal agriculture are least willing to accept changes to these animals but are perhaps more ambivalent towards the creation of new products such as lab meat. Additionally, people most supportive of animal agriculture may be least willing to accept the use of genetic technologies on these animals. Some people supportive of agriculture may still recognise the welfare and environmental consequences of certain practices and thus view the BC and IA as “the wrong kind of solution to the problem” (Devolder 2021; p 15); for these participants, lab meat may be perceived as a way of reducing harms and a viable alternative for the market.

Participants who ate meat and those who did not were similarly supportive of the blind chicken and insentient animal scenarios (t_254_ = 1.85; *P* = 0.0649; and t_245_ = 0.017; *P* = 0.7863), but non-meat-eaters were more supportive of laboratory meat than were meat-eaters (averaging 4.8 [± 0.34] versus 3.5 [± 0.12] on the seven-point scale; t_239_ = 3.62; *P* = 0.0004).

People who believed that the technology failed to respect dignity of life were more likely to reject its use; this result held true for the blind chicken (slope = 0.85, SE = 0.06, t_255_ = 13.49; *P* < 0.0001), and insentient animal (slope = 0.75, SE = 0.07, t_246_ = 10.07; *P* < 0.0001) scenarios, and especially so for the laboratory meat scenario (slope = 1.02, SE = 0.06, t_240_ = 16.41; *P* < 0.0001) (Figure 1[b]). The interaction suggests that although few participants in the cultured meat scenario felt that it was disrespectful, those who did feel this way were especially affected by this concern.

Violations of dignity might be interpreted in various ways, including as a reflection of excessive instrumentalisation. In Swiss animal welfare law, the concept of dignity is used as a focal point for understanding the ethical appropriateness of interventions in the lives of non-human animals, including animal experimentation (Bolliger 2015). Part of the definition of dignity used to inform practices includes violations of dignity through “humiliation, excessive instrumentalisation, and substantial interference with an animal’s appearance or abilities” which includes interacting with animals as objects rather than respecting their own interests and natures (Bolliger 2015; p 338). Cataldi (2002) and Humphreys (2016) argue that the moral weight of dignity does not require any psychological states within the animal (i.e. animals need not desire dignity or be aware of any degradation). From this perspective, sentient and non-sentient animals alike may be seen to have their dignity violated.

Humphreys (2016) notes that transgressions of dignity take place when an action holds no benefit to the subject; however, the aim of reducing animal suffering was central to the justification for the genetic modifications explored in this study. The concerns in the current study around dignity suggest that some participants did not believe the scenarios were of actual benefit to the animals. We found that participants were less supportive of technologies when they did not perceive these to be beneficial to animals. Overall, 20.9% of participants believed that the treatments benefited the animals; this percentage was lowest for the blind chicken scenario (8.6%) and highest for the lab meat scenario (32.2%), with the insentient scenario perceived as intermediate (22.6%). Participants who perceived the technology to benefit animals were more supportive of its use for all three scenarios (3.0 [± 0.3] vs. 1.4 [± 0.1], *F*_1,255_ = 22.99; *P* < 0.0001; 2.7 [± 0.2] vs. 1.3 [± 0.1], *F*_1,246_ = 37.46; *P* < 0.0001; and 3.7 [± 0.2] vs. 2.2 [± 0.1], *F*_1,240_ = 40.57; *P* < 0.0001, for the blind chicken, insentient animals, and lab meat scenarios, respectively). The purported purpose of genetic technologies (e.g. to benefit human health or food system actors) is known to influence acceptance (Hudson *et al.* 2015; Ritter *et al.* 2019), such that technology framed as benefiting some aspect of social good (including the welfare of the animals) is generally more supported. Further work is required to better understand the type of benefits required, and what type of evidence of benefits people find compelling.

Participants who believed that the technology was available on the market showed more support for the creation of blind chickens (slope = 0.39, SE = 0.12, t_255_ = 3.21; *P* = 0.0015) and for the creation of insentient animals (slope = 0.28, SE = 0.10, t_246_ = 2.81; *P* = 0.0053), but there was no evidence of a relationship for laboratory meat (slope = 0.11, SE = 0.12, t_240_ = 0.93; *P* = 0.3525) (Figure 1[c]). That people were more likely to support technology when they believed it was already on the market, could reflect a sense of complacency; believing that a technology was already prevalent may have resulted in participants believing that there was little to be done to change the situation.

### Qualitative results

Qualitative analysis of the open-ended text responses was intended to help understand participant rationale regarding their responses to the three scenarios. For each scenario, participants were first told that animals typically experience certain harms as part of conventional agriculture. For example, in the blind chicken scenario condition participants were told that, “Chickens housed in groups can peck one another, sometimes resulting in injury or even death. To deal with this problem, farmers generally remove part of the beak of young chicks without the use of anaesthetic (i.e. pain-control).” They were then told that the scenario was intended to mitigate this harm. In the case of the blind chicken scenario, participants were told, “Alternatively, it is possible to use genetic modification to create chickens that are born blind. Blind chickens live similarly to sighted chickens but are less likely to engage in harmful pecking, eliminating the need to partially remove their beaks.” Thus, our study design was intended to encourage participants to balance the perceived harms associated with current practices against any new harms associated with the proposed technology. Given this framing, we expected participants to use a type of trade-off logic in their responses; expressions of or trade-off thinking was included as our first *a priori* theme. Our second *a priori* theme was intended to capture the rejection of this framing, specifically by participants rejecting both the new technology and the *status quo.* In the sections that follow we illustrate both themes with participant quotes (citation also includes the country of the participant (Canada = CA, United States = US), participant number, and the scenario this participant was assigned to (Blind chickens = BC, Insentient animals = IA, Cultured meat = CM).

### Trade-off thinking

The trade-off thinking theme included comments that displayed a cost-benefit analysis of scenarios, where perceived harms and benefits were weighed against one another, for example, in their considerations of the different options offered, or other issues of importance for participants. In all scenarios, participants who supported the proposed solution (i.e. blind chickens, creating insentient animals, and growing cultured meat) mentioned reducing harms associated with the initial problem they were presented with (e.g. feather-pecking, and stress related to handling, transport, and slaughter). For example, one participant [CA 217 IA] stated: “*I don’t think humans will stop eating animals and animal products anytime soon, at least not in most countries, so it’s important to do what we can to prevent harm to those animals.*”

Responses to the laboratory meat scenario often mentioned that this option was preferable to harming animals. For example, one participant [CA 36 CM] said, “*I think* [cultured meat] *could be the right option because that way animals are not being harmed.*” Another [US29 CM] stated, “*I’m an animal advocate and vegetarian. I’d rather people eat artificial stuff than torment animals.*” Other participants mentioned that cultured meat products were desirable alternatives because they were better for the environment, or for feeding a growing population. In all these cases participants seemed comfortable within the dichotomy used to frame the issue and felt that one of the response options was acceptable. For example, CA 44 CM stated, “*Perhaps lab processed* [meat] *would be better for environment.*”

Some participants applied trade-off thinking but expressed some unease in doing so. For example, one participant [US 4 IA] explained: “*I can completely see why this would be beneficial, but at the same time, it feels very ethically wrong.*” Similarly, US 266 IA stated that, “*It would be better to create something that doesn’t know or feel what is about to happen*”, but still questioned whether it was right “*to mess with living creatures’ lives?*” Another participant [US 317 BC] commented that [creating blind chickens] “*Seems bad but I guess it’s for a good reason. Rather have blind chickens than them being pecked to death by each other.*” These responses appear to illustrate unease in accepting seemingly logical proposals to alter animals to reduce animal suffering in animal agriculture, when the moral intuition is that adopting such proposals is wrong (Thompson 2008; Palmer 2011).

A number of participants expressed discomfort with the premise and the limited range of response options. For example, one individual [CA 427 BC] questioned the morality of systems that would lead to such choices, stating “*This is what mass-production does. I’m sure there would be a fight in any circumstance that 100 creatures were packed into a tight cage*”, but concluded that, given the seemingly dichotomous response options of either accepting the *status quo* or the new technology, that they would “*go with the* [new technology]”. This participant recognised that the genetic application could provide immediate relief but their response points to the inability of this choice to engage with systemic issues (Murphy & Kabasenche 2018). Some participants called for something to be done for animals in agricultural systems (e.g. stating that it is “…*a travesty that animals suffer*” [CA 20 IA]), that there are likely other options available to address suffering (e.g. “*we could also just outlaw the small, confined quarters*” [CA 78 BC]).

As discussed by Macnaghten (2004), certain types of framing (e.g. the dichotomous presentation of choices existing within the *status quo*) “may inadequately accommodate both the range and the potential novelty of ethical concerns raised by animal biotechnology” (p 537), including concerns related to the instrumental use of animals and “wider unease about science, about technological modernity, and about hubris.” (p 533). Our results suggest that some participants engaged with the choice between the new technology and the *status quo*, but others baulked at the dichotomy, for example, explaining that their acceptance of the new technology was conditional on these two choices being the only ones available. This result suggests that proponents of technology may benefit by framing issues in a dichotomous manner, but this approach can also create a false sense of consensus. In a forced choice scenario, participants may simply be picking the ‘least bad’ option; this does not indicate that either option is considered acceptable or attractive. This line of reasoning would support the use of questions in future studies that explicitly ask participants to rate the acceptability of both options independently, rather than indicate if one is more acceptable than another. Even without this option, some participants in the current study explained in their qualitative response that both options were unacceptable, as we describe in the following section.

### Rejecting both response options

Some animal ethicists have argued that, despite the moral unease associated with dis-enhancements like creating individual animals without sight or sentience, these technologies should be adopted given that the *status quo* results in suffering of “actual, living and breathing animals” (Thompson 2008; p 311). Participants in the current study sometimes appeared unwilling to accept this logic and rejected both options provided, for example, by rejecting production methods that would make these technologies necessary. One participant [US 54 BC] in the blind chicken scenario said that the proposed solution to the problem of feather-pecking still involved “*inhumane treatment of the animal*” and questioned how this “*could… possibly be better for the animal?*” Another [CA 148 BC] simply stated that, “*Either approach is wrong.*” These participants seemed to question the contention that these interventions could be of actual benefit to the animals. Similarly, Murphy and Kabasenche (2018) ask “whether the changes being proposed in animal disenhancement are truly for the sake of the animals, or whether they represent an attempt not to change most of the other features of the system” (pp 228–229).

Some participants disagreed that interventions like creating blind chickens and insentient animals were the only or most viable options. For example, CA 278 BC said “…*I believe there has to be another way*”; a sentiment echoed by CA 289 BC who suggested that the problem “*Could be dealt with* [using] *a different option.*” With respect to the issue of feather-pecking, several participants suggested improved rearing conditions. For example, CA 215 BC stated that a “*better solution would be not to house chickens in such close confines rather than developing needless genetic modifications.*” Similarly, CA 159 BC wrote, “*Don’t raise chickens in that sort of environment. The problem can be prevented!*” Murphy and Kabasenche (2018) argue that if “the starting point for justifying disenhancement is the reduction of suffering, there are many ways we could achieve that” (p 228), including modifying environments. Comments from our participants express what Devolder (2021) identified as the “wrong kind” (p 3) of solution to address suffering. This author looked to the side-effects of two choices to reduce animal suffering: using genome editing to create pigs resistant to a respiratory disease, or giving them more space to reduce disease transmission, to illustrate why the latter is the preferable option. Increasing space provides a solution to more than one problem; it decreases disease transmission *and,* by decreasing stressful interactions that arise from dense housing, it increases animals’ well-being. While some participants were not convinced of the proposed dichotomy or felt that there were preferable options available for alleviating suffering, others specifically called for systemic change. For example, CA 364 IA wrote that “*Animals shouldn’t have to change; people and their methods should.*” Similarly, CA 67 CM asked that “*great minds come up with a better way*” of doing things. Thus, at least some people were unwilling support these types of interventions do so because they do not see these as acceptable approaches to the problem. Indeed, while there were important differences between the blind chicken and insentient animal scenarios, most notably that the latter scenario described animals with no welfare to be considered, participants responded to these interventions as similarly undesirable perhaps because they considered both to be the wrong type of solution to the underlying ethical issue.

Harfeld *et al.* (2016) speak to the elements of industrial agriculture that prevent people from seeing both the moral relevancy of animals and the systemic flaws including how animals are spoken about in agricultural systems (i.e. as extensions of production machinery), how they are housed and treated, and the incremental measures that are sought to improve their lives including those tethered to increases in production efficiency. Devolder (2021) suggested that a desire to reduce a sense of complicity is one reason why some people may reject interventions like the ones our participants encountered, when viable alternative ways to address issues exist. According to this way of thinking, these narrowly framed solutions make people morally complicit in the wrongness that the system is seen to perpetuate.

Schultz-Bergin (2014) makes a distinction between approaches that examine rational choices in a non-ideal world (e.g. where *status quo* production systems continue to exist because of resistance to change within the industry), versus more ideal-world thinking that includes rejecting *status quo* conditions and advocating for changes in production methods to avoid creating welfare problems in the first place. Visioning more ideal systems allows us to question the “assumptions that gave rise to the problems in the first place.” (p 204). Thus, it is important to distinguish between approaches that seek to confront the acceptability of a technology more narrowly in the context of a non-ideal, non-changing world, versus more generally “reflecting on what sort of world we want to create and the role such idealisation plays in evaluating our actual world” (p 16). If a person’s ideal is a world without industrial agriculture, for example, then genetic interventions like creating blind chickens and insentient animals are unlikely to be perceived as helpful. From a psychological perspective, reasoning styles differ between people, based on what values they hold, with consequences for decision-making (Billet 2021). For people with reasoning styles governed by sacred values, a deontological style of reasoning means that trade-offs offered between the sacred (in this case animals) and the non-sacred (production efficiency) will be viewed as taboo and morally impermissible (Tetlock 2003). In contrast, those holding trade-off reasoning styles, are more likely to be influenced by instrumental values (Atran 2016). In addition, our results suggest that framing genetic technology as the only alternative to the suffering experienced by animals within the *status quo* system of agriculture is viewed as too narrow by some participants, in part, “because actions occur within a context, and this context bears on our understanding” of actions (Schultz-Bergin 2014; p 7).

According to Macnaghten (2004), people contextually develop ethical positions towards animals and how they are treated, related to how technologies symbolise and reflect “specific societal values and assumptions” (p 534), including values and assumptions about what kinds of relationships society ought to expect between humans and animals. Some participants in our study suggested that both the human conduct and the human-animal relationship represented in the scenario were ethically problematic. These participant comments correspond well with discussion in the academic literature; Kramer and Meijboom (2021) and Sandøe *et al.* (2014) discuss animal integrity as a morally relevant source of unease when considering the creation of new strains of animals through genetic interventions, like blind chickens. For example, CA 66 IA suggested that “*Just from a moral position, it should be inhumane to change how animals behave just to suit our needs.*” Similarly, CA 91 BC stated that creating blind chickens is “*ethically wrong and* [a] *selfish thing to do*”, and US 460 BC stated that this is a “*self-serving way to raise chickens, depriving them of their sight.*” Murphy and Kabasenche (2018) suggested that disenhancing animals reinforces principles of domination. Thompson (2008) described some objections to genetic disenhancements as rooted in concerns about the virtue of those who would do this, saying performing such genetic modifications can be seen as evidence of the “vices of pride, of arrogance, of coldness and of calculating venality” (p 314). Some participants in the current study also referred to such character flaws (e.g. “*Genetically altering animals to make them easier to kill is just plain evil.*” [US 284 IA]).

Interestingly, the qualitative theme of rejection of both options was observed only twice for the cultured meat scenario; the rarity of this theme for this scenario might be due to participants viewing this technology as a way of rejecting conventional animal agriculture.

## General discussion and Conclusion

Participants in the current study responded to the scenarios we presented by considering the broader context of the production systems. Both quantitative and qualitative results illustrate the importance of factors that seem to extend beyond considerations associated with the trade-offs of technologies that were offered to participants. For example, some participants considered whether the scenario reflects excessive instrumentalisation of animals, whether it benefits the animals themselves, and if it is seen as respecting the dignity of life. These results suggest that questions relating to dignity and beneficiary, as well as to the instrumental use of animals, should be considered in future work on public attitudes towards technologies applied to animals. Thompson (2008) suggested that framing the issues related to genetic alteration of animals as a problem of moral character distracts people from harms to animals, but the responses from participants in our study suggest that the moral character of the system and opportunity for restructuring are important considerations. Seen in this light, framing arguments about technology only around alleviating suffering associated with current production methods may be insufficient, as this side-steps a broader discussion of rearing systems and the morality of those responsible for these systems. Previous studies examining attitudes to genetic modification of farm animals often do not offer options beyond acceptance of either the technology or the *status quo* (McConnachie *et al.* 2019; Ritter *et al.* 2019; Yunes *et al.* 2019; Ly *et al.* 2021; Naab *et al.* 2021); to our knowledge, no study to date has specifically examined the rejection of the existing response options as a theme in participant qualitative responses. The qualitative responses from participants in this study suggest the need to include other options in discussions around new technologies related to the production of animal products, including new ways of raising animals for food and non-animal alternatives.

Our study has several limitations. We surveyed US and Canadian participants using census matched quotas (age, gender, and income), but our sample should not be considered fully representative as some demographic factors were not considered and willingness to participate in the survey introduces at least some bias (Paolacci & Chandler 2014). Further, participants from this region should not be considered representative of people from other regions.

One possible source of confusion for participants is that some may have believed that modifications were to existing, living individual animals (i.e. believing that previously sighted or sentient individuals would now be made blind or insentient), and did not realise that instead it was the progeny that would be born blind or insentient. Our description of the scenario attempted to avoid this confusion, for example, specifying that the insentient animals would be “born without the capacity to feel pain, fear, or pleasure.” Future work should specifically ask participants if they believed that capacities were removed from existing individuals or understood that young would be born this way.

Some of the wording used to describe the scenarios may have caused confusion for participants (although none reported this in their qualitative comments). For example, in the formulation of the blind chicken scenario, we mention that blind chickens live similarly to sighted chickens. By this we meant that the birds live in the same housing conditions. We did not mean that these birds have the same quality of life as their sighted counterparts. Similarly, we described the cultured meat as being “the same as that from animals raised for food”; by this we meant the same product. We did not mean that the process used to create this product was similar in these two cases.

We used *a priori* themes related to trade-off thinking and rejection of this framing as these related directly to our aim of understanding how participants think about these issues. Other themes related to genetic interventions and animal welfare are already well-covered in the literature, but the use of an alternative coding approach, such as an open method, may have provided more context and helped explain participant responses (Williams & Tami 2019).

### Animal welfare implications

Thompson (2008) noted that social repugnance to proposed interventions for solving animal welfare issues in industrial farming could provide impetus to change circumstances for animals.

Our study shares the perspectives of people who, faced with contentious methods of creating new animals and animal products to address issues within current production systems, sometimes reject these options, and instead call for system-level change. Thus, our study design illustrates a method for understanding when people may be willing to accept changes within a system versus when they will call for more transformative changes in animal agriculture. Work that frames issues as trade-offs within a system may discourage transformational thinking, limiting our understanding of people’s concerns and thus also limiting the development of policy and practices that improve the lives of farmed animals.
